# Supplementary figures and images for: Visualizing synaptic plasticity in vivo by large-scale imaging of endogenous AMPA receptors
Source: eLife. 2021 Oct 18;10:e66809. doi: 10.7554/eLife.66809 (PMC8616579; doi:10.7554/eLife.66809)

Fig. 1 Source Data 1

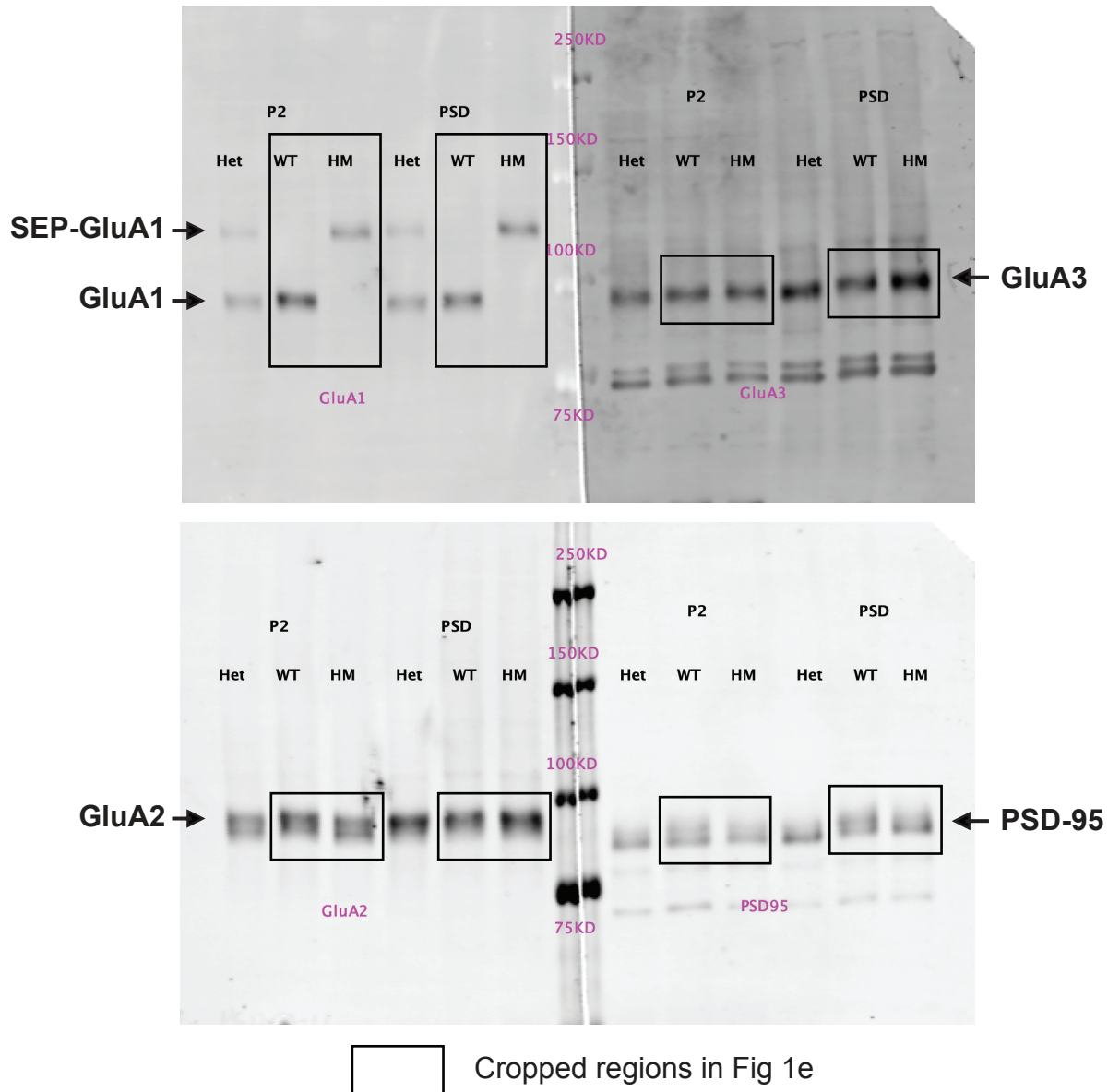

Supplement: Figure 1—source data 1. — Blots were stained for GluA1 (top left), GluA2 (bottom left), GluA3 (top right), and PSD-95 (bottom right). Lanes corresponding to homozygous SEP-GluA1 (HM), heterozygous SEP-GluA1 (Het), and wild-type C57BL6 (WT) mice are indicated, for both the P2 and postsynaptic density (PSD) fractions. Size ladder is indicated in magenta. Black boxes indicate cropped regions of representative blots in Figure 1e. [file elife-66809-fig1-data1.pdf]

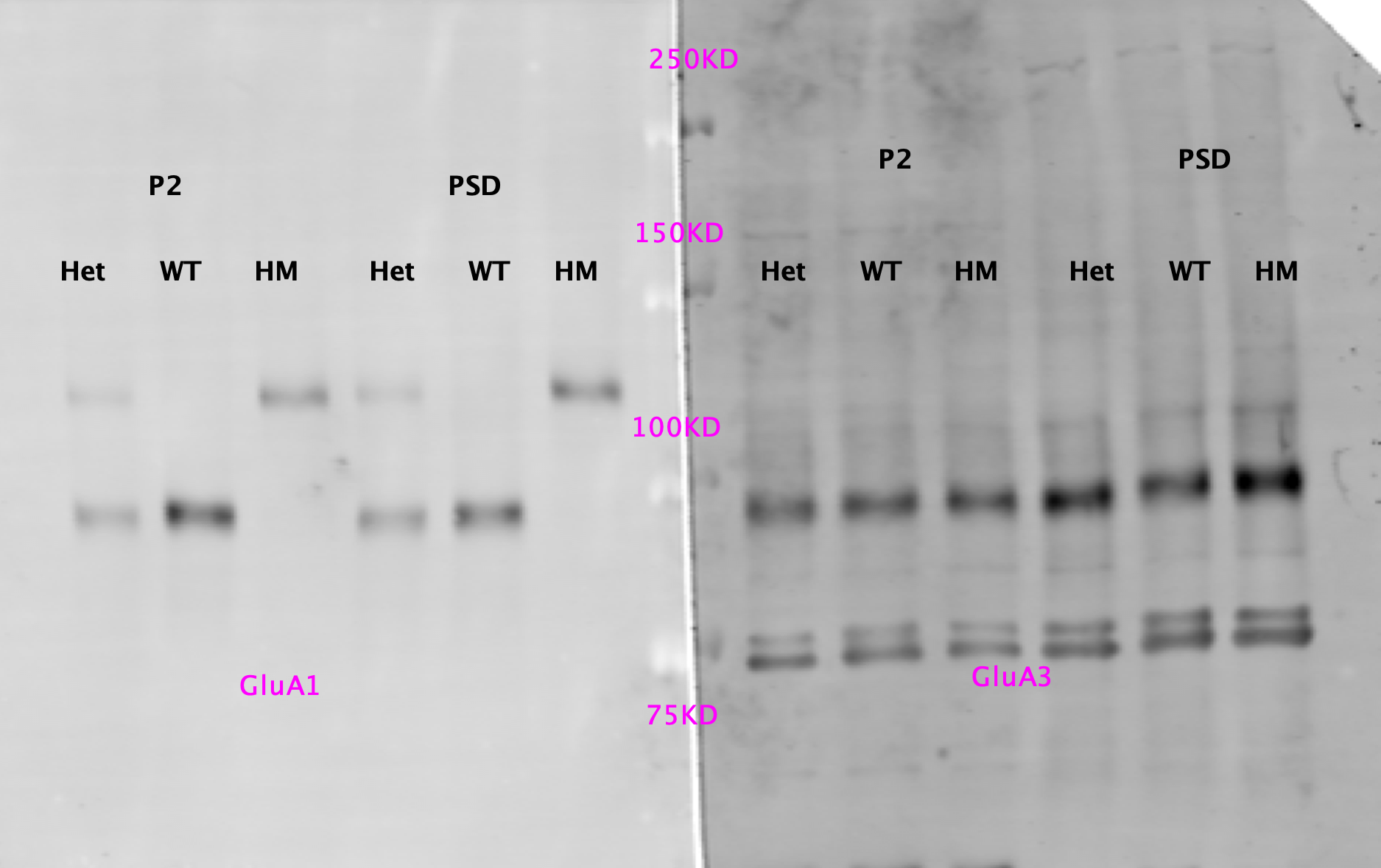

Supplement: Figure 1—source data 2. [file elife-66809-fig1-data2.zip › Figure 1 - Source data 2.tif]

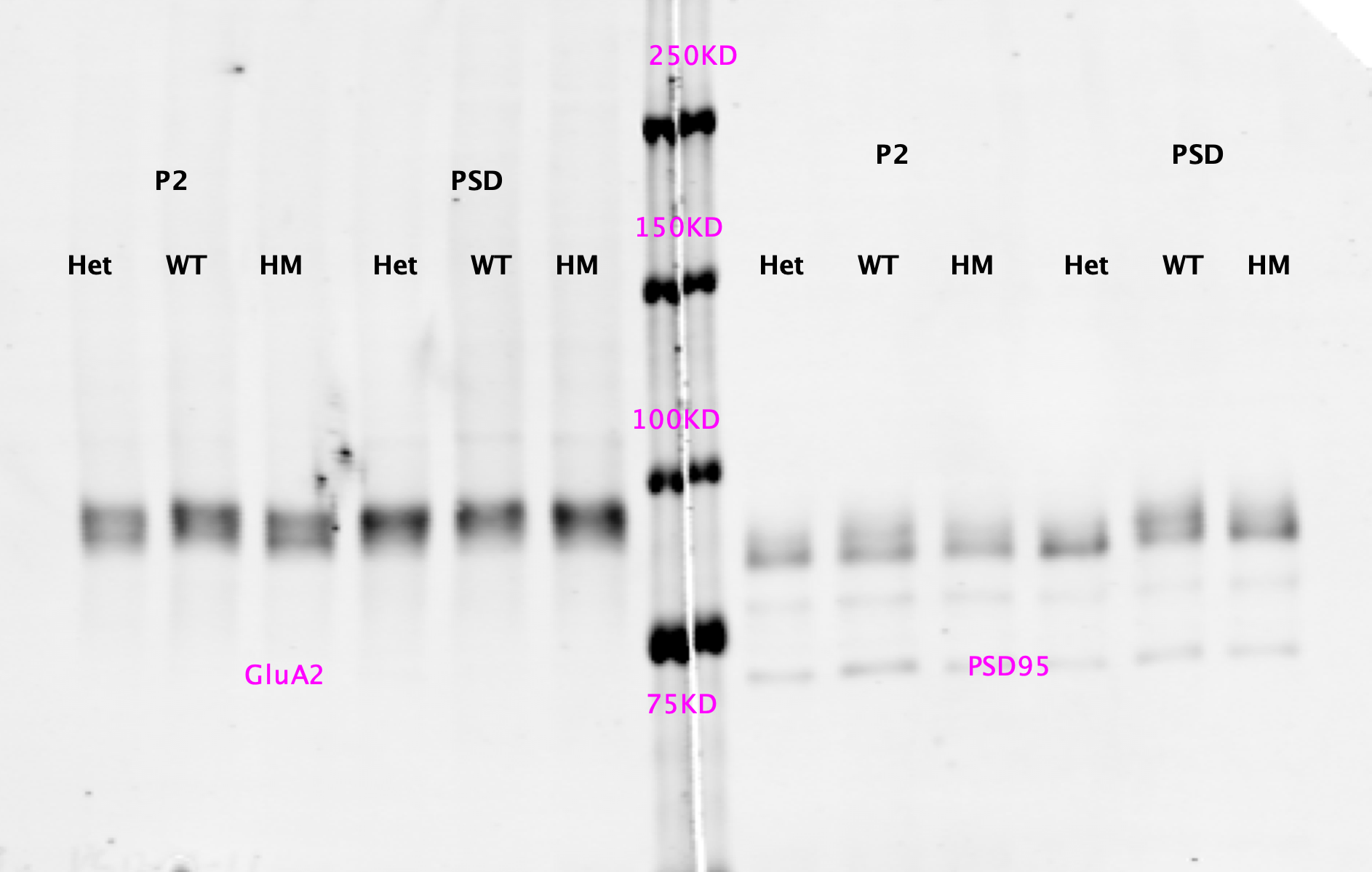

Supplement: Figure 1—source data 3. [file elife-66809-fig1-data3.zip › Figure 1 - Source data 3.tif]

Fig. 3 Source Data 1

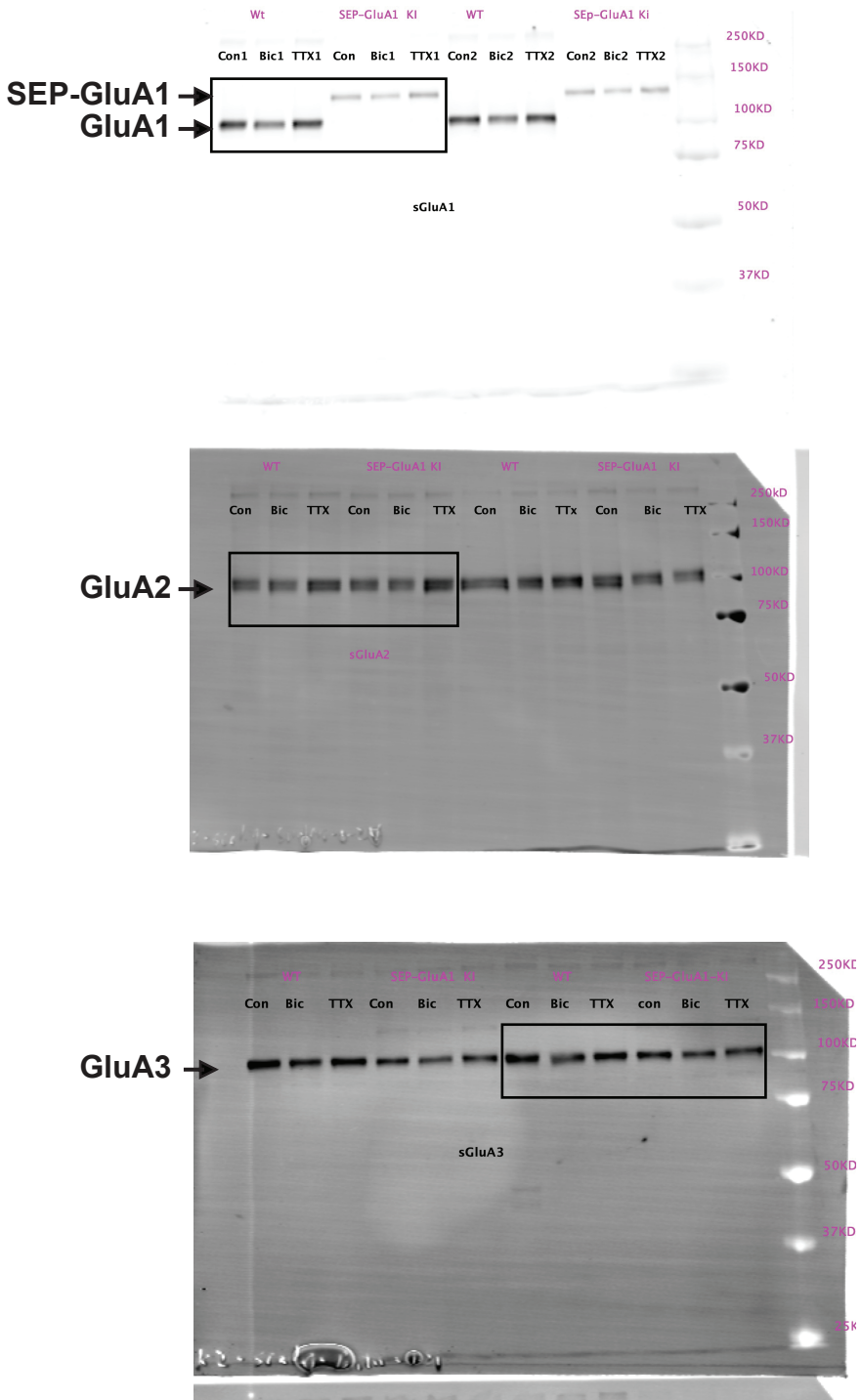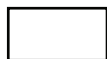

Cropped regions in Fig. 3a

Supplement: Figure 3—source data 1. — Blots were stained for surface expression of GluA1 (top), GluA2 (middle), and GluA3 (bottom). Lanes corresponding to control (Con), bicuculline (Bic), and tetrodotoxin (TTX) conditions are indicated, for both wild-type (WT) and SEP-GluA1 mice. Size ladder is indicated in magenta. Each blot contains two biological replicates. Black boxes indicate cropped regions of representative blots in Figure 3a. [file elife-66809-fig3-data1.pdf]

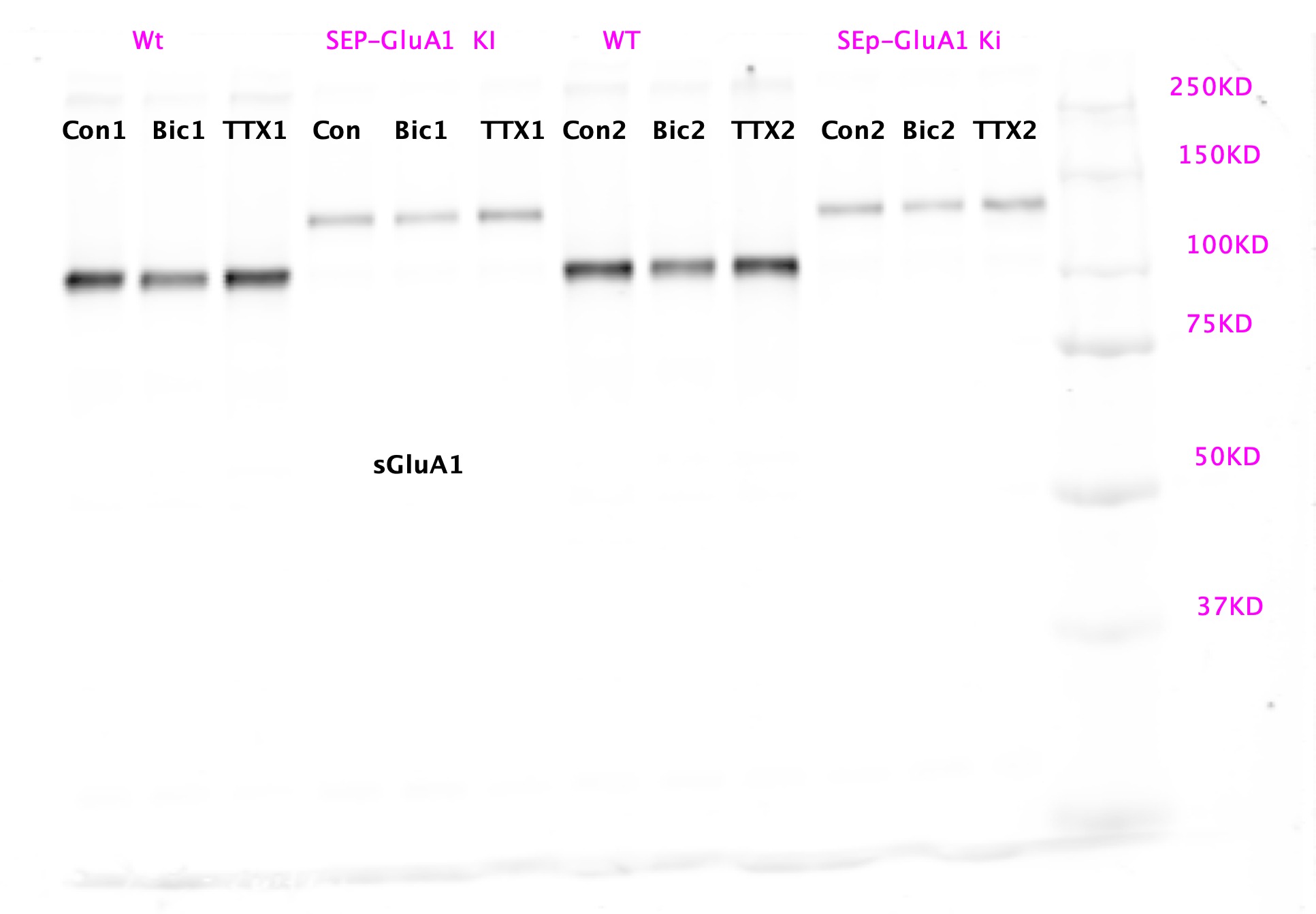

Supplement: Figure 3—source data 2. [file elife-66809-fig3-data2.zip › Figure 3 - Source data 2.tif]

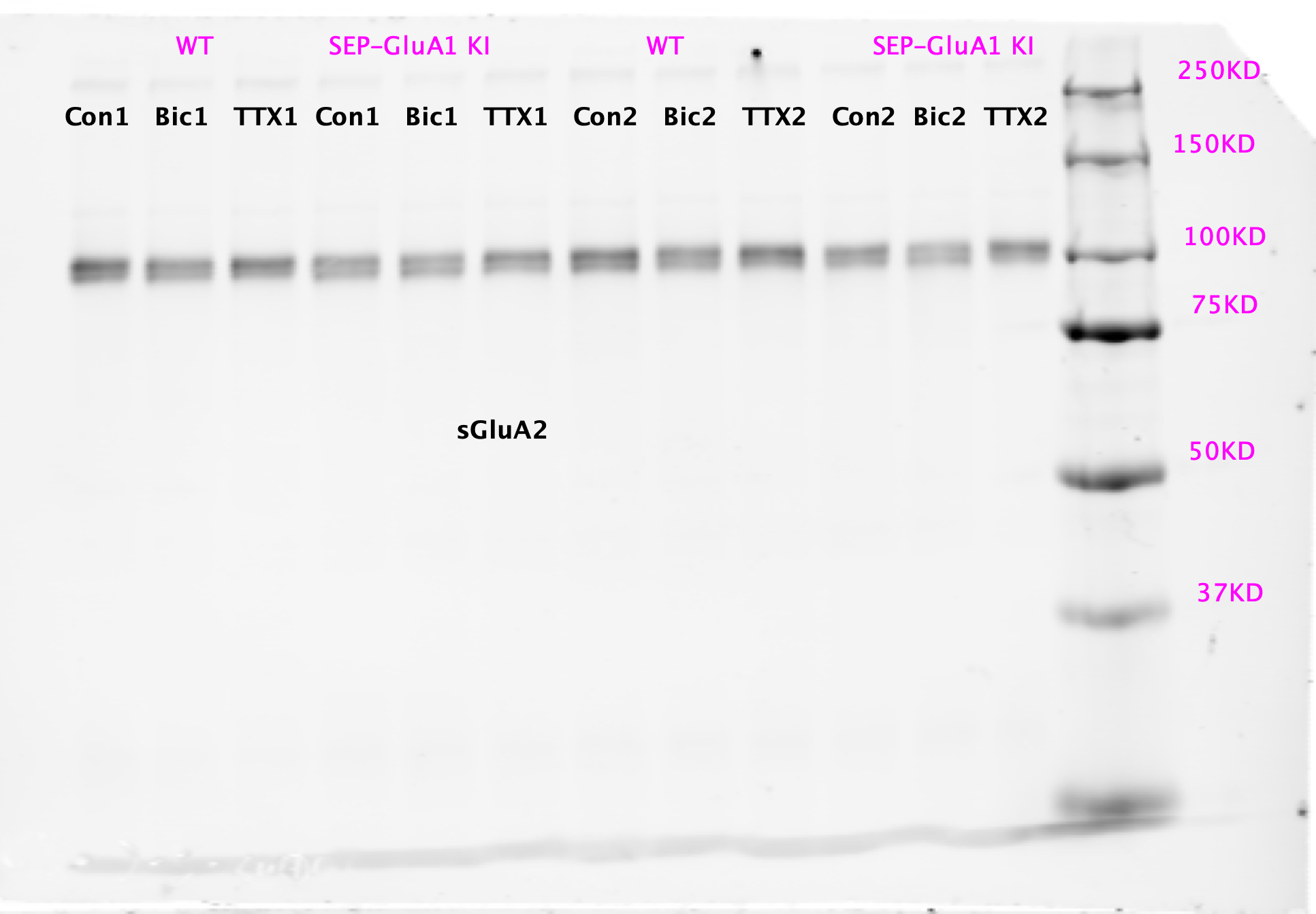

Supplement: Figure 3—source data 3. [file elife-66809-fig3-data3.zip › Figure 3 - Source data 3.tif]

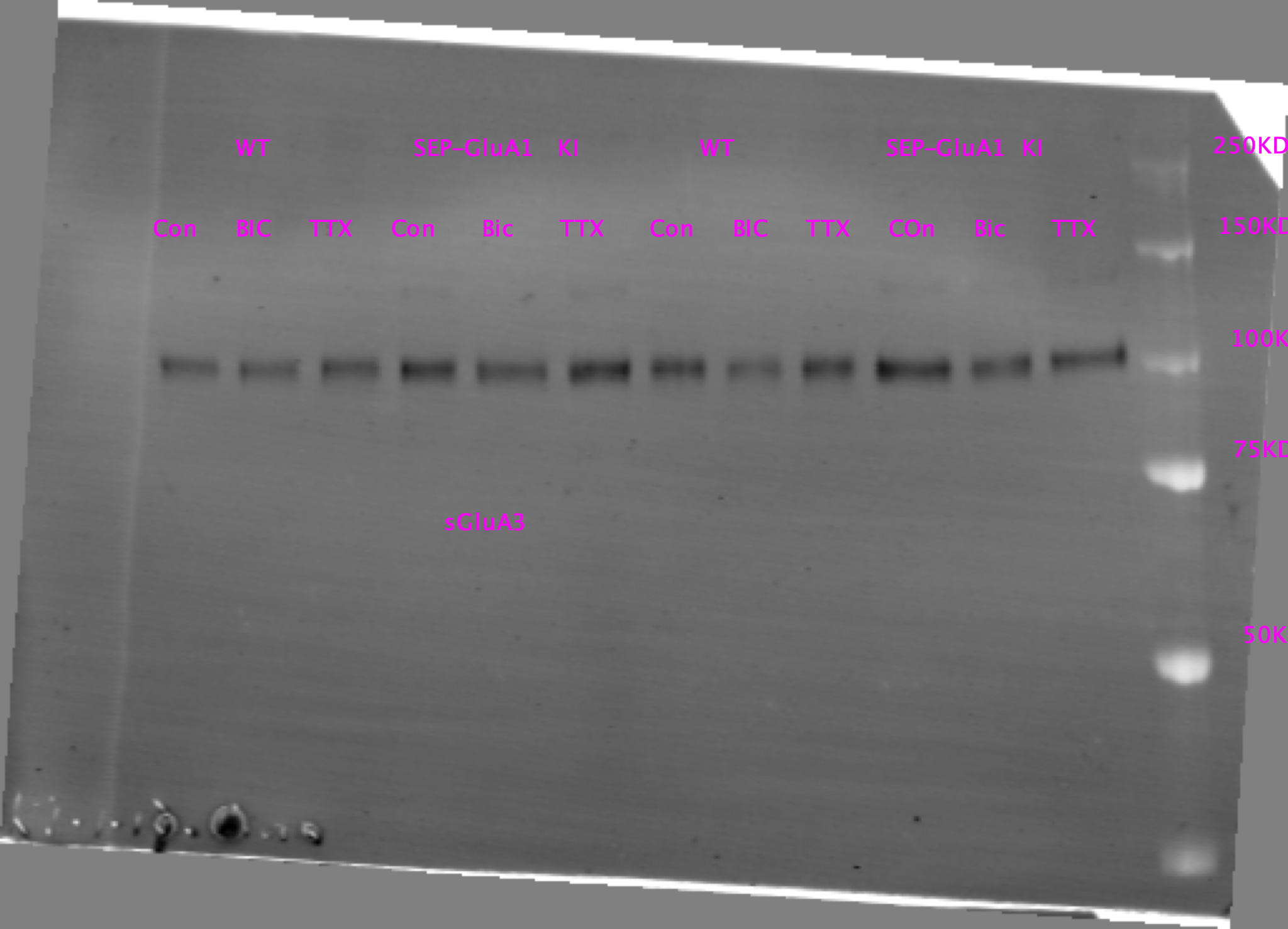

Supplement: Figure 3—source data 4. [file elife-66809-fig3-data4.zip › Figure 3 - Source data 4.tif]
